# Whole-genome sequences of *Odocoileus hemionus* deer adenovirus isolates from deer, moose and elk are highly conserved and support a new species in the genus *Atadenovirus*

**DOI:** 10.1099/jgv.0.000880

**Published:** 2017-08-15

**Authors:** Myrna M. Miller, Todd E. Cornish, Terry E. Creekmore, Karen Fox, Will Laegreid, Jennifer McKenna, Marce Vasquez, Leslie W. Woods

**Affiliations:** ^1^​ University of Wyoming, Wyoming State Veterinary Laboratory, 1174 Snowy Range Road, Laramie, WY 82070, USA; ^2^​ Wyoming Game and Fish Department, Wyoming State Veterinary Laboratory, 1174 Snowy Range Road, Laramie, WY 82070, USA; ^3^​ Colorado Division of Parks and Wildlife, Wildlife Health Program, 4330 Laporte Ave, Fort Collins, Colorado 80521, USA; ^4^​ California Animal Health and Food Safety Laboratory, School of Veterinary Medicine, 620 West Health Science Dr., 620 West Health Science Dr, Davis, CA 95616, USA

**Keywords:** cervid, adenovirus, *Atadenovirus*, moose, elk, deer

## Abstract

We present the first complete genome sequence of *Odocoileus hemionus* deer adenovirus 1 (OdAdV-1). This virus can cause sporadic haemorrhagic disease in cervids, although epizootics with high mortality have occurred in California. OdAdV-1 has been placed in the genus *Atadenovirus*, based on partial hexon, pVIII and fibre genes. Ten field isolates recovered from naturally infected mule deer (*Odocoileus hemionus*), white-tailed deer (*Odocoileus virginiana*) and moose (*Alces alces*) from Wyoming, black-tailed deer (*Odocoileus hemionus columbianus*) from California, and Rocky Mountain elk (*Cervus elaphus nelsoni*) from Colorado and Washington state were sequenced. The genome lengths ranged from 30 620 to 30 699 bp, contained the predicted proteins and gene organization typical of members of genus *Atadenovirus*, and had a high percentage of A/T nucleotides (66.7 %). Phylogenic analysis found that the closest ancestry was with ruminant atadenoviruses, while a divergence of the hexon, polymerase and penton base proteins of more than 15 % supports classification as a new species. Genetic global comparison between the 10 isolates found an overall 99 % identity, but greater divergence was found between those recovered from moose and elk as compared to deer, and a single variable region contained most of these differences. Our findings demonstrate that OdAdV-1 is highly conserved between 10 isolates recovered from multiple related cervid species, but genotypic differences, largely localized to a variable region, define two strains. We propose that the virus type name be changed to cervid adenovirus 1, with the species name Cervid atadenovirus A. Sequence data were used to develop molecular assays for improved detection and genotyping.

## Abbreviations

A.a., moose; BAdV, bovine adenovirus; C.e., elk; EBM-2, endothelial cell basal medium-2; ITR, inverted terminal repeat; ML, maximum likelihood; OAdV, ovine adenovirus; OdAdV-1, Odocoileus hemionus deer adenovirus; O.h., mule deer; O.h.c, black-tailed deer; ORF, open reading frame; O.v, white-tailed deer; SNP, single nucleotide polymorphism.

## Introduction

Adenoviruses have worldwide distribution and infect vertebrates of all types. They are typically well adapted to specific host species and infection is frequently subclinical, except for in young or immunocompromised individuals [1]. Exceptions occur, such as turkey haemorrhagic disease and canine infectious hepatitis [2]. Exceptions also occur for host-specific infections, as recently shown for a primate adenovirus (titi monkey adenovirus) with the potential to infect humans [3]. Adenoviruses of humans have been found to be extremely hardy and may remain in the environment for long periods in sheltered areas [4, 5], providing a source of infection for susceptible individuals.

Members of the family *Adenoviridae* have been divided into five genera [6]. The genus *Atadenovirus* was recently added in 2002 to include adenoviruses that were previously assigned to the genus *Mastadenovirus*, but varied significantly based on genomic size (20 % smaller), smaller structure, genes and gene arrangement, and codon usage biased to A/T-rich triplets – hence the genus name *Atadenovirus* [7, 8]. More recently, numerous diverse genus members have been described from reptile hosts that lack the A/T bias [9–12]. In general, a low GC content is associated with a recent host-switching event, suggesting that the original genus ancestor was from a squamate host [13, 14]. Atadenoviruses lack homologous genes for EIA, E3, V, IX and viral-associated RNA [7, 15], and there is an especially A/T-rich noncoding region of unknown function near the 3′ end of the genome of ruminant atadenoviruses [16]. This new genus contains seven species (Virus Taxonomy: 2016 Release. International Committee on Taxonomy of Viruses), with viruses that infect a diverse range of animals, including avian, ruminant, reptile and marsupial hosts [9, 17–20]. A complete or partial genome sequence has been determined for the genus members, including ovine adenovirus 7 (species *Ovine atadenovirus D*, OAdV-D; type species of the genus) [16], bovine adenovirus 4 [8] (within species *Bovine atadenovirus D*, BAdV-D), bovine adenovirus 6, duck adenovirus 1 [21], lizard adenovirus 2 [9], possum adenovirus 1 [17], psittacine adenovirus 3 [19] and snake adenovirus 1 [12, 14].

*Odocoileus hemionus* deer adenovirus 1 (OdAdV-1) is a member of the genus *Atadenovirus* that can cause systemic vasculitis with the potential for high fatality rates, and has been partially sequenced [22, 23]. Sporadic epizootics of adenovirus haemorrhagic disease in mule deer (*Odocoileus hemionus,* O.h.) and black-tailed deer (*Odocoileus hemionus columbianus,* O.h.c., a subspecies of mule deer) have been reported in California [24, 25]. White-tailed deer (*Odocoileus virginiana,* O.v.) and moose (*Alces alces*, A.a.) have also been found to be susceptible [26, 27]. Experimental challenge studies have induced fatal disease in young fawns, but mild or undetected infections in yearlings [28–30]. Lesions include pulmonary edema and haemorrhagic enteropathy, and may be similar to the haemorrhagic disease caused by the orbiviruses: bluetongue virus and epizootic haemorrhagic disease virus. Naturally occurring cases of adenovirus infection in wildlife are typically discovered as mortalities, but cases of mild disease with recovery are not likely to be observed. Serological testing to determine prevalence has proved challenging due to cross-reactions with related ruminant viruses [31], demonstrating the need for improved antibody detection assays. Little is known of the epidemiology and pathogenesis of OdAdV-1, or whether genetic variation in the virus contributes to variable outcomes of localized sporadic disease or large epizootics.

The purpose of our study was to sequence the complete genome of OdAdV-1, analyse the phylogenic relationship with other members of the genus *Atadenoviruses*, and determine the genetic variability between 10 isolates recovered from related host species in multiple states. Adenovirus recovered from naturally infected animals was identified by traditional methods of virus isolation, electron microscope imaging and PCR. Whole-genome sequences of field isolates collected from mule deer, white-tailed deer, black-tailed deer, elk and moose over 17 years were compared to each other by multisequence alignment and protein-coding regions were identified. The phylogenic relationship between isolates was examined using the whole-genome nucleotide sequence, and the relationship to other related adenoviruses was assessed based on amino acid sequences coding for the penton base, polymerase and hexon proteins. Sequence data were used to develop sensitive and specific assays for diagnosis and genotyping.

## Results

### Genome size and organization

**Fig. 1.**
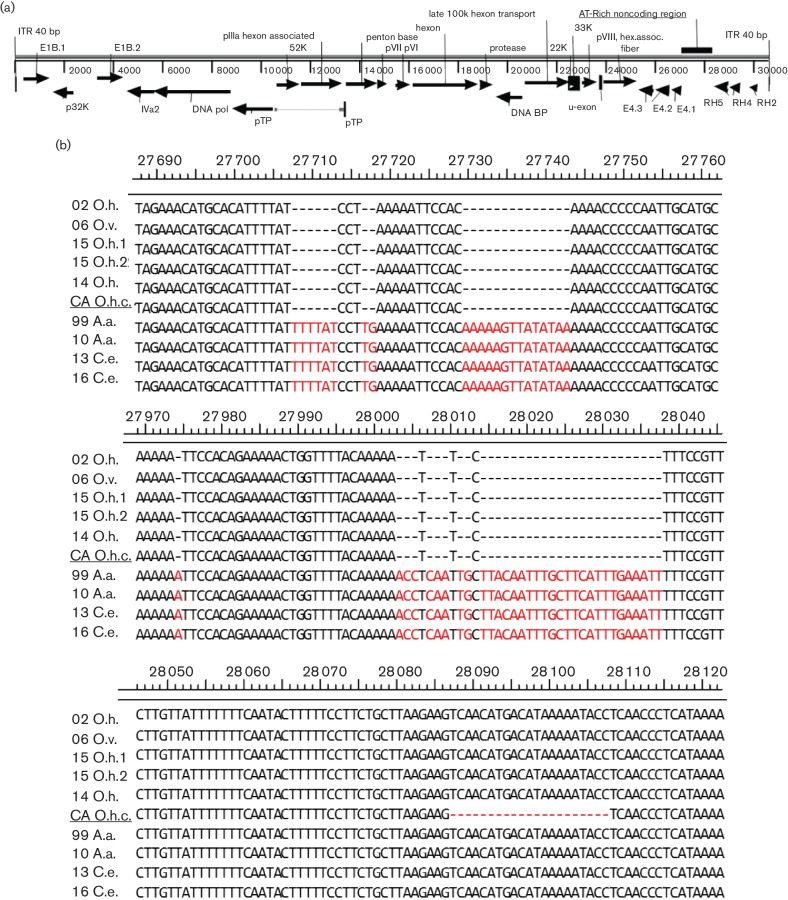
The genome organization of OdAdV-1 is depicted schematically (a). Potential ORFs were identified using blastx search to identify homologous proteins in GenBank. The genome sequences between the 10 isolates were highly identical (99 %), but had a variable region within the A/T-rich noncoding region (NCR). Multiple sequence alignment of the 10 isolates from the variable region is shown (b). This region contained two deletions totalling 54 nt (22 nt+32 nt, O.h. and O.v.) or three deletions totaling 75 nt (O.h.c.) compared to moose and elk (A.a., C.e.).

The genomes of 10 OdAdV-1 isolates were fully sequenced and analysed. The genetic length varied between 30 620 and 30 699 bp, with 40 bp inverted terminal repeats (ITR) and an overall A/T content of 66.7 %. The gene arrangement and A/T-rich content are typical of the genus *Atadenovirus* from ruminant hosts. The genome arrangement of the predicted proteins is presented schematically in Fig. 1(a). Genus-specific findings, from 5′ to 3′, include: a p32K homologue in the EI genomic region, no E3 homologue in the region between pVIII and fibre genes, lack of homologues for V and IX, the presence of special E4 genes (E4.1) and a set of right-hand genes (RH2, 4 and 5) [9, 32]. Also present is a 1300 bp especially A/T-rich (72.6 %) noncoding region unique to the genus located between RH5 and E4.1 that has been described in ruminant atadenoviruses [16]. An unexpected finding was an apparent complete duplicate of the E1B gene located between the left-hand ITR and p32K, which is shown as E1B.1 in Fig. 1(a). This open reading frame (ORF) codes for a 379 aa protein that has 50 % identity with the second E1B gene (381 aa protein, E1B.2 in Fig. 1(a)). The E1B.2 gene is in the expected location and is >99 % identical to the current OdAdV-1 sequence in GenBank (AAF13267). These two E1B proteins have 46–52 % identity to E1B proteins of other ruminant atadenoviruses based on blastp search results. The whole-genome sequences of seven of the isolates have been submitted to GenBank and assigned the accession numbers KY468402- KY468407 and KY748210 (Table 1).

**Table 1.** Ten *Odocoileus hemionus* deer adenovirus isolates were sequenced as part of the study. Isolates were recovered from diagnostic cases received from 1999 to 2016 Host species included Rocky Mountain elk (*Cervus elaphus nelsoni*, C.e.), moose (*Alces alces*, A.a.), mule deer (*Odocoileus hemionus,* O.h.), black-tailed deer (*Odocoileus hemionus columbianus*, O.h.c.) and white-tailed deer (*Odocoileus virginiana* O.v.).

Isolate	Host	Year	Location	Age/diagnosis	GenBank accession
99A.a	A.a.	1999	Wyoming	Calf/adenovirus mortality	KY468407
10A.a.	A.a.	2010	Wyoming	Calf/adenovirus mortality	KY468404
13C.e.	C.e.	2013	Washington	Juv/apparent health	KY468405
16C.e.	C.e.	2016	Colorado	Adult/adenovirus related mortality	KY468406
02O.h.	O.h.	2002	Wyoming	Yearling/adenovirus mortality	KY468402
14O.h.	O.h.	2014	Wyoming	Fawn/adenovirus mortality	
15O.h.1	O.h.	2015	Wyoming	2 weeks/adenovirus mortality	
15O.h.2	O.h.	2015	Wyoming	4 months/adenovirus mortality	
06O.v.	O.v.	2006	Wyoming	1.5 years/adenovirus mortality	KY468403
CAO.h.c.	O.h.c.	1998	California	Adenovirus mortality	KY748210

### Two strains based on a single variable region

**Fig. 2.**
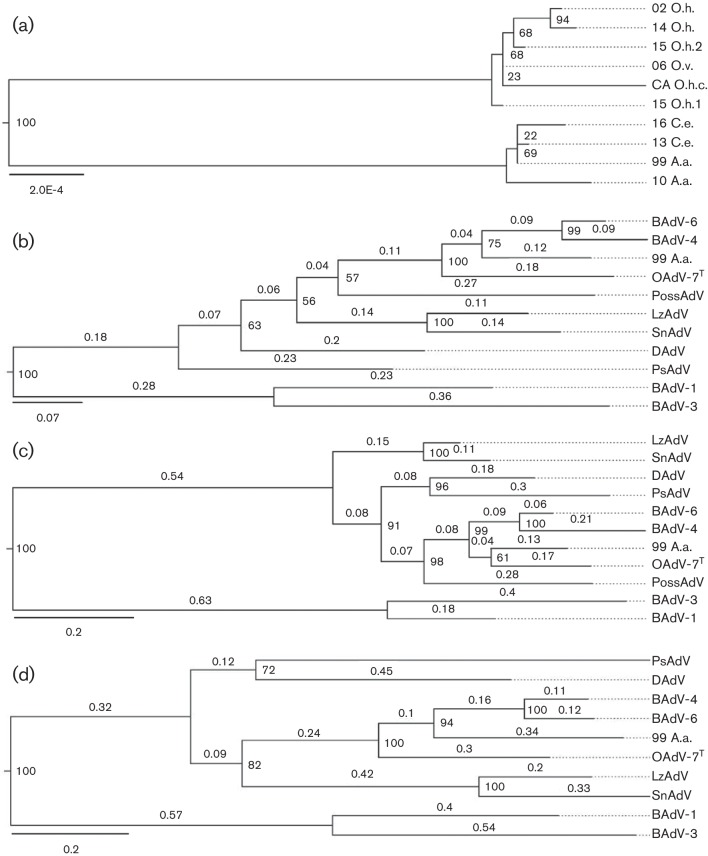
Maximum-likelihood phylogenic trees depicting the relationship between the 10 OdAdV-1 isolates in this study based on whole-genome sequences (a). Maximum-likelihood trees estimating the relationship between OdAdV-1 and the other atadenoviruses based on full amino acid sequences of the penton base (b), hexon (c) and polymerase proteins (d). Trees were constructed using PhyML in SeaView version 3.2 with a BioNJ starting tree and bootstrap values calculated from 1000 pseudo-replicates. A single OdAdV-1 sequence (99 A.a.) was used in (**b)–(d**) due to isolate identity in these proteins. Atadenoviruses: ovine adenovirus 7 (OAdV^T^, type species), duck adenovirus 1 (DAdV), psittacine adenovirus 3 (PsAdV), possum adenovirus 1 (PossAdV), lizard adenovirus 2 (LzAdV), snake adenovirus 1 (SnAdV), and bovine adenovirus 4 and 6 (BAdV-4 and 6). Representative ruminant viruses from the genus *Mastadenovirus* were included as outliers, bovine adenoviruses 1 and 3 (BAdV-1 and 3). Bootstrap values and branch lengths (b)–(d) are given. The accession numbers for the protein reference sequences retrieved from GenBank are listed in Table 2.

The multiple sequence alignment of the 10 isolates found the genetic sequence is highly conserved, with greater than 99 % sequence identity. However, a variable region is present within the A/T-rich noncoding region between the RH5 and E4.1 genes (Fig. 1(a)). This region was longest in the isolates from elk and moose (*N*=4). The isolates from mule deer and white-tailed deer (*N*=5) had two deletions totalling 54 nt (22 plus 32 nt), and the California black-tailed deer isolate had an additional 21 nt deletion, as shown in the multiple sequence alignment (Fig. 1(b)). Largely based on this region, the genome length varied and resulted in two distinct strains. However, even outside of this region, over 100 single nucleotide polymorphisms (SNP) segregated in the same pattern, with 12–29 SNPs between members of the same strain, and 186 SNPs between members of the opposite strain. This difference resulted in an overall percentage identity of 99.9 % within a strain and 99.7 % between strains. The phylogenic tree based on whole-genome sequences of the ten isolates demonstrates two distinct clades, Fig. 2(a).

### Phylogeny inference

The partial OdAdV-1 sequences currently in GenBank (AF198354, AF198355, AF198356 and AF361168) are 99 % identical to the nucleotide sequences of our isolates. Maximum-likelihood (ML) phylogenic estimation comparing OdAdV-1 with other genus members based on full-length predicted amino acid sequences for the penton base, hexon and polymerase proteins found that the closest relationship was with other ruminant atadenoviruses, OAdV-7, BAdV-4 and BAdV-6 (Fig. 2(b,d)). The phylogenic relationship was also analysed using partial amino acid sequences of the hexon and pol proteins so that BAdV-7 could be included, and this resulted in the same topology and similar branch lengths compared to the other ruminant atadenoviruses (data not shown). The most conserved genes were the structural proteins, hexon and penton base, with greater divergence being evident in the amino acid sequence for the polymerase protein, as has been found previously in phylogenic analysis for the genus [19].

### Diagnostic and genotyping PCR

**Fig. 3.**
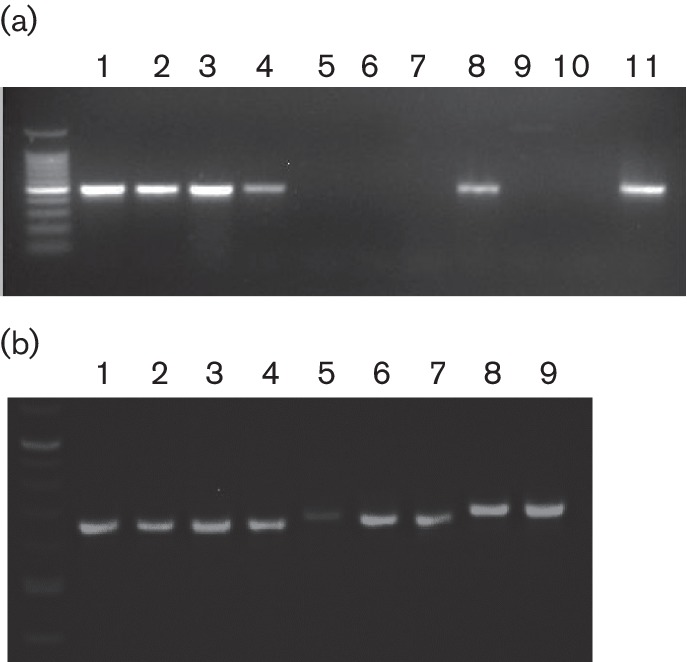
Primers specific for OdAdV-1 were designed for a diagnostic PCR and a genotyping PCR. Assays were run using DNA extracted from additional diagnostic cases that were positive for OdAdV-1 or other viral agents to test for specificity. (a) The diagnostic PCR amplified a 502 bp amplicon, a 100 bp molecular weight marker, and OdAdV-1 from mule deer (lanes 1, 3), elk (lane 2), pronghorn (lanes 4, 8) and moose (lane 11). No amplification product was produced with samples positive for bovine, canine, or ovine adenovirus of undetermined type (lanes 5–7), chlamydophila from a mule deer (lane 9), or the no template control (lane 10). (b) The genotyping PCR was designed to span part of the variable region and produced amplicons of 680, 701, or 733 bp, depending on the presence or absence of deletions: lanes 1–4, California black-tailed deer (680 bp due to 53 bp deletion); lanes 5–8, pronghorn, white-tailed deer, mule deer, (701 bp due to 32 bp deletion); and lane 8–9, mule deer with the moose/elk variation (733 bp with no deletions). Traditional Sanger sequencing using forward and reverse primers confirmed the expected sequence changes.

A PCR was developed to aid in the sensitive and specific detection of OdAdV-1 DNA in diagnostic material. Our use of a previously published assay [28] was found to give false positive reactions when applied to samples from pronghorn (*Antilocapra americana*), with Sanger sequencing of the amplicon finding pronghorn satellite DNA sequences. The new assay was able to detect OdAdV-1 in all of the species in the study, as well as pronghorn, with amplicons verified by Sanger sequencing using forward and reverse primers (Fig. 3(a)). To test for primer specificity, DNA from samples that were positive for bovine adenoviruses 1 and 4, canine adenovirus 2, ovine adenovirus (type not determined), bluetongue virus, epizootic haemorrhagic disease virus, pestivirus (bovine and pronghorn), chlamydophila sp. and cervid herpesvirus were not detected by the assay. A genotyping PCR was also designed using primers flanking the second and third deletions found in the variable region. This assay produced bands of 680, 701, or 733 bp, depending on the strain (Fig. 3(b)). This assay was used on DNA from an additional eight diagnostic cases positive for OdAdV-1 with amplicons run on a gel for size comparison and sequenced to verify the results. Of these eight, virus DNA from four additional black-tailed deer from a California epizootic contained the same changes as the isolate from the California black-tailed deer in our study. Virus DNA extracted from mule deer and pronghorn tissues had the deer strain sequence, and samples from two other mule deer had the longer sequence found in moose and elk virus from our study (Fig. 3(b)).

## Discussion

The whole genome sequences in this report have 99 % identity with the partial OdAdV-1 sequences in GenBank, confirming that it is the same virus that was previously reported. The complete genome sequence contains homologous genes and a gene arrangement that is typical of the genus *Atadenovirus*, which confirms the previous classification in this genus [22, 23]. Of interest is an apparent duplication of the E1B gene in the region between the left-hand ITR and the p32 gene. The evolutionary and functional significance of this duplication remains to be determined. The exceptionally low GC content (33.3 %) of the genome may indicate that this virus is the result of a relatively recent host-switching event [13]. Phylogenic analysis showed that the closest ancestry was with other ruminant atadenoviruses, based on penton base, hexon and polymerase protein amino acid sequences (Fig. 2 (b–d)). The species demarcation criteria in the family *Adenoviridae* is 5–15 % divergence in the DNA polymerase amino acid sequence [6]. The divergence of greater than 15 % for the hexon and penton base proteins, and greater than 30 % for the polymerase protein, of OdAdV-1 compared to other genus members indicates this is a new species within the genus. This is supported by challenge studies where cross-infection of bovine adenoviruses and OdAdV-1 failed to produce infections in the opposite host [33]. The nearly identical isolates recovered from multiple related host species within the family Cervidae suggests that the species name should be Cervid atadenovirus A, and that the type name should be cervid adenovirus 1.

A distinct variable region was identified in the genus-unique A/T-rich noncoding region (Fig. 1(b)). Based largely on differences in this region, our isolates separated into two distinct clades (Fig. 2(a)). The function of this region is currently unknown [16]. These different clades segregated with host species, with virus from elk and moose being distinct from those from deer. However, the number of samples in this study is not sufficient to support a host-specific variation hypothesis. Indeed, using the genotype PCR and sequencing the amplicons from additional diagnostic cases, OdAdV-1-positive DNA from two mule deer mortalities in Wyoming in 2015 was found to have the elk/moose variation (Fig. 3(b)). These two cases were part of a cluster of mule deer adenovirus haemorrhagic disease mortalities in a region with large populations of moose and elk, providing potential for close contact. It is also of interest that the isolate recovered from a black-tailed deer from a large California epizootic [25] had an additional 21 bp deletion in this same region (Fig. 1(b)). The genotyping PCR found the same deletion in an additional four California cases from epizootic events (Fig. 3(b)). Large epizootics of adenovirus haemorrhagic disease resulting in thousands of mortalities have not been observed outside of California and Oregon, suggesting that the additional deletion might be significant. Sequencing of additional cases from California unrelated to large outbreaks would be useful in determining whether this additional deletion is likely to be related to pathogenesis, and this would need to be confirmed with challenge studies.

Most of the isolates (9/10) were recovered from animals succumbing to adenovirus haemorrhagic disease. However, the prevalence and distribution of OdAdV-1 and the potential for mild disease, chronic infections, and shedding are important unanswered questions. Studies have been limited due to the challenges of few diagnostic tools and the difficulty of sampling populations of wild animals. The isolate that came from an apparently healthy elk (Table 1, 13 C.e.) indicates that, at least transiently, OdAdV-1 can be detected in some healthy animals. Pathogenicity studies based on experimental infections have produced serious disease in fawns, but mild or inapparent disease in yearlings or adult animals [28–30]. Naturally occurring cases of adenovirus haemorrhagic disease are also more frequent in neonates and the young, but can occur animals of all ages. Factors affecting immune competence, such as stress and nutrition status, are likely to play a role in disease outcome. Adenovirus can survive for long periods outside of a host, and this, combined with the presence of susceptible animals, likely accounts for many cases of disease.

Our whole-genome sequence data and global comparison between 10 isolates identified a variable region distinguishing distinct genetic strains of OdAdV-1. The contribution of genetic variation to the epidemiology of adenovirus haemorrhagic disease remains to be tested. Further studies will be needed to determine the distribution and host preference of these strains, and whether the genetic variation contributes to variable pathogenesis. Complete genetic information and improved assays for diagnosis and genetic variation testing will be valuable tools for such pathogenicity and epidemiology studies.

## Methods

### Animals

Seven of the 10 isolates in the study were recovered from naturally occurring wildlife adenovirus haemorrhagic disease mortalities investigated by the Wyoming Game and Fish Department and submitted to the Wyoming State Veterinary Laboratory for diagnostic testing (Creekmore *et al*., manuscript in preparation). Of the remaining three isolates in the study, one was recovered from elk tissues received from the Colorado Division of Parks and Wildlife from an animal with ruminal ulcers as well as other multisystem lesions [34], one was recovered from an apparently healthy elk (unpublished finding) from the state of Washington that was a negative control from an unrelated study [35] and one was recovered from a black-tailed deer from the 1993/94 California epizootic [25]. These cases are listed in Table 1. These adenoviruses were identified by PCR, virus isolation, EM imaging and in some cases the detection of typical intranuclear bodies by histopathology.

### Isolation and culture of mule deer umbilical vein endothelial cells

Umbilical cords from third-trimester twin mule deer foetuses were harvested aseptically and placed into 199E medium (Cellgro, Manassas, VA, USA) supplemented with 5 % ultimate grade FBS (Seradigm, Providence, UT, USA) and 2× penicillin/streptomycin (Lonza, Walkersville, MD, USA; 200 units ml^−1^ and 200 µg ml^−1^). The umbilical veins were dissected out and rinsed with PBS (Cellgro). One end of each vein was clamped and the veins filled with 1 mg ml^−1^ collagenase (Sigma-Aldrich, St Louis, MO, USA) in PBS, then the the other end was clamped and incubated for 5 min while resting in PBS. The veins were rinsed with collagenase, and then the digest was repeated, with incubation for 1 h. The veins were flushed with 199E into a centrifuge tube and the cells collected by centrifugation at 250 ***g*** for 8 min. The cells were resuspended in endothelial cell basal medium-2 (EBM-2; Lonza) supplemented with endothelial cell growth factors (EGM-2 single quots; Lonza) and 15 % FBS and incubated in cell culture-treated flasks at 37 °C, 5 % CO_2_ until confluent. Cells were processed using 0.05 % Trypsin-EDTA (Gibco, Grand Island, NY, USA) in PBS, and frozen at a low passage number (5–6) using dimethylsulfoxide (11 %, Sigma-Aldrich) in FBS. The white-tailed deer umbilical vein endothelial cells used in some cases were similarly prepared. Cultured cells were confirmed to be free of adenovirus, pestivirus and *Mycoplasma* species by PCR.

### Virus isolation and purification

Tissue pools (a combination of two or more: lung, liver, kidney and spleen) from diagnostic cases submitted to the Wyoming State Veterinary Laboratory were homogenized in 1 : 5 vol of Bovarnick’s media (0.218 M sucrose, 3.8 mM KH_2_PO_4_, 7.2 mM K_2_HPO_4_, 4.9 mM glutamic acid; Sigma-Aldrich) and applied to 60-70 confluent mule deer umbilical vein endothelial cells or white-tailed deer umbilical vein endothelial cells drained of media. After 1 h adherence, inoculum was removed and cells were refed with complete media: EBM-2, 4 % FBS and 1× penicillin/streptomycin (Lonza; 100 units ml^−1^ and 100 µg ml^−1^) at 37 °C, 5 % CO_2_. Cultures were observed daily for cytopathic effect (CPE). Wells with no CPE after 7 days were frozen and then thawed, after which the supernatant was passed onto new cells for two blind passages. For sequencing, virus from the second to fifth passage was grown in 150 cm^2^ flasks to >/=90 % cytopathic effect, frozen, thawed and clarified by centrifugation at 5000 ***g*** for 20 min prior to centrifugation through a 20 % sucrose cushion at 107 000 ***g*** for 12 h.

### DNA extraction

Pellets were resuspended in 400 ul PBS and the DNA extracted using filter columns (Qiagen Blood and Tissue Kit, Valencia, CA, USA) according to the manufacturer’s directions. The DNA concentration was estimated by fluorimetric measurement (Qubit, ThermoFisher Scientific, Waltham, MA, USA).

### Sequencing and analyses

Shotgun libraries were prepared using the Hyper Library Construction kit (Kapa Biosystems, Wilmington, MA, USA) and sequenced on Illumina MiSeq with a Nano V2 kit (High-Throughput Sequencing and Genotyping Unit, Roy J. Carver Biotechnology Center/WM Keck Center, University of Illinois at Urbana-Champaign). Sequences were assembled *de novo* using the Ray Assembler program (Ray version 2.3.1, publicly available software) [36] and for mapping assembly MIRA version 4 was used [37]. In all cases, assembly of the next-generation data produced a single contiguous sequence of the expected size. These were examined using Tablet [38] for quality, SNP confirmation and depth of coverage. The average coverage depth for the 10 isolates was >2000 (214–11 480). PCR and traditional Sanger sequencing covering over 10 % of the genome (3115 bp) further checked the accuracy of the sequences. ORFs were identified and annotated using SeqBuilder, Lasergene 13 (DNASTAR, Madison, WI, USA), and the predicted transcription units were examined for homologous proteins in GenBank via Basic Local Alignment Search Tool (blast), using the blastx and blastp queries [39].

### Phylogenic analysis

The phylogenic relationship between the isolates in our study was estimated, and it was also determined for OdAdV-1 and other atadenoviruses and representative bovine viruses from the genus *Mastadenovirus* as outliers. Sequences were aligned using BioNJ [40] and ML phylogenic trees (PhyML version 3.1) were constructed using programs in the Seaview version 4.6.1 software package [41, 42]. Distance was calculated with Kimura’s two-parameter model [43] and compared to the ML-generated tree branch length using programs in Seaview. The phylogenic tree comparing the 10 isolates to each other was based on whole-genome nucleotide sequences. Phylogenic trees comparing OdAdV-1 to other adenoviruses were based on the predicted complete amino acid sequences for the penton base (450 aa), hexon (911 aa) and polymerase (1076 aa) proteins. The accession numbers for the reference sequences retrieved from GenBank are listed in Table 2.

**Table 2.** The reference sequences used in phylogenic tree construction were retrieved from GenBank for full coding sequences for the penton base, hexon and polymerase proteins All seven species within the genus *Atadenovirus* are represented, and two ruminant mastadenoviruses are included as outliers.

Adenovirus type (species)	Penton base aa	Hexon aa	Polymerase aa
Bovine adenovirus 4 *(D)*	NP_077393	NP_077397	NP_077389
Bovine adenovirus 6	YP_007347002	YP_007347006	YP_007346998
Ovine adenovirus 7^T^ *(D)*	NP_659519	NP_659523	NP_659515
Possum adenovirus 1 *(A)*	AF249332	AAL73247	Not available
Duck adenovirus 1 *(A)*	NP_044706	NP_044710	AP_000080
Psittacine adenovirus 3 *(A)*	YP_009112720	YP_009112724	YP_009112716
Lizard adenovirus 2 *(A)*	YP_009051658	YP_009051622	YP_009051654
Snake adenovirus 1 *(A)*	YP_001552251	YP_001552255	YP_001552247
Bovine adenovirus 1 *(A)*	YP_094036	YP_094039	YP_094032
Bovine adenovirus 3 *(B)*	AP_000028	NP_ 046323	AP_000026

T, type species of the genus *Atadenovirus.*

### PCR assays

Whole-genome sequence data were used to develop molecular assays for diagnosis, genotyping and validation of next-generation sequencing results. The primers were *in silico* tested for specificity using National Center for Biotechnology Information (NCBI) Primer-blast [44], and the diagnostic primers were *in vitro* tested for cross-reactions with common bovine and cervid viruses. Sanger sequencing of the PCR amplicons using forward and reverse primers (GENEWIZ, South Plainfield, NJ, USA) was used to verify the amplicons. The primer pairs listed in Table 3 were used with Platinum PCR Supermix (Life Technologies, Carlsbad, CA, USA). A reaction volume of 25 ml contained 1 ul each 10 uM F and R primers, 3 ul DNA template and 20 ul Supermix. The thermocycler conditions were 94 °C 2 min, (94 °C 15 s, 62 °C 1 min, 72 °C 30 s) × 40, 72 °C 2 min.

**Table 3.** Whole-genome sequence data were used to design PCR assays for diagnosis, genotyping and validation of assembly data by Sanger sequencing

Primer	Sequence (5′– 3′)	Position*	Purpose, amplicon size (bp)
Ad Aa F	CCGCATTCAGGCCACTTCCATT	29 915–29 936	Diagnosis, 502 bp
Ad Aa R	TGCTGCGTCTGAACACTAATA	30 396–30 416	
Ad 6 L F	TTACCCAAAATTCCCTATCCA	27 769–27 789	Genotyping, 680, 701 and 733
Ad 6 L R	TTGCGTGTTACTAATGCTGTTCG	28 477–28 501	
Ad13/14 F1	GAACTTCCCAGACCATTTACC	3 093–3 113	Validation, 505
Ad13/14 R1	AGGCATTTCCGTTTTACTCTG	3 577–3 597	
Ad 13/14 F2	GTGGCCTAGTAATTTTCGTCTGC	4 107–4 129	Validation, 653
Ad 13/14 R2	TTCCTCCTAGTTTGATTCCTGTGT	4 736–4 759	
Ad 13/14 F3	AAGCGCTACACTGCAATAACAATC	14 737–14 760	Validation, 776
Ad 13/14 R3	GACCAGCTGAAACCACCTCTC	15 492–15 512	

*Based on the nucleotide sequence of OdAdV-1 99A.a. (GenBank KY468407.)

### Nucleotide sequence accession numbers

Sequence data were submitted to GenBank with accession numbers KY468402- KY468407 and KY748210.
